# Involvement of the Ligninolytic System of White-Rot and Litter-Decomposing Fungi in the Degradation of Polycyclic Aromatic Hydrocarbons

**DOI:** 10.1155/2012/243217

**Published:** 2012-07-04

**Authors:** Natalia N. Pozdnyakova

**Affiliations:** Institute of Biochemistry and Physiology of Plants and Microorganisms, Russian Academy of Sciences, 13 Prospekt Entuziastov, Saratov 410049, Russia

## Abstract

Polycyclic aromatic hydrocarbons (PAHs) are natural and anthropogenic aromatic hydrocarbons with two or more fused benzene rings. Because of their ubiquitous occurrence, recalcitrance, bioaccumulation potential and carcinogenic activity, PAHs are a significant environmental concern. Ligninolytic fungi, such as *Phanerochaete chrysosporium, Bjerkandera adusta*, and *Pleurotus ostreatus*, have the capacity of PAH degradation. The enzymes involved in the degradation of PAHs are ligninolytic and include lignin peroxidase, versatile peroxidase, Mn-peroxidase, and laccase. This paper summarizes the data available on PAH degradation by fungi belonging to different ecophysiological groups (white-rot and litter-decomposing fungi) under submerged cultivation and during mycoremediation of PAH-contaminated soils. The role of the ligninolytic enzymes of these fungi in PAH degradation is discussed.

## 1. Introduction

**Figure 1:**
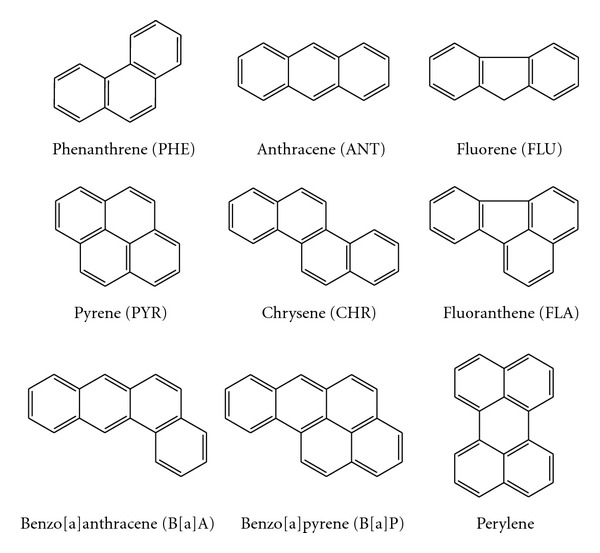
Chemical formulas of some 3-, 4- and 5-ring PAHs [http://www.chemport.ru/].

The use of fossil fuels for energy and raw material in the past century has led to widespread environmental pollution. Among these pollutants are polycyclic aromatic hydrocarbons (PAHs), which are considered a potential health risk because of their possible carcinogenic and mutagenic activities [1]. PAHs consist of benzene analogs having two or more aromatic rings in various alignments (Figure 1). Most of the low-molecular-weight PAHs (up to three aromatic rings) are very toxic [2], and most of the high-molecular-weight PAHs (four and more aromatic rings) are highly mutagenic, teratogenic, and carcinogenic for humans and animals [3]. PAHs are compounds of great environmental significance and are considered by the Environmental Protection Agency (USA) and other national institutions to be of toxicological relevance [http://www.defra.gov.uk/Environment/consult/airqual01/11.htm].

The general and scientific interest in the fate of PAHs in the environment and their microbial degradation, especially of higher-molecular-weight PAHs consisting of more than four rings, is based on their carcinogenic and mutagenic properties. Many reviews are available on different aspects of PAH degradation [4–6]. Several fungi are known to have the property of degradation PAHs. The degradation of these compounds by ligninolytic fungi, including white-rot and litter-decomposing fungi, has been intensively studied. They produce extracellular enzymes with very low substrate specificity, making them suitable for degradation of lignin and different low- and higher-molecular-weight aromatic compounds [6].

Investigations into the microbial bioconversion of PAHs has shown that wood- and litter-decay fungi are efficient degraders of these organopollutants [7–11]. They can mineralize PAHs with four and more condensed aromatic rings, in contrast to bacteria and soil fungi. They also can metabolize both individual PAHs and their complex mixtures, such as creosote [12–23]. The toxicity of PAHs underlies the use of creosote, a PAH mixture, as a fungicidal wood preservative. However, many fungi, including white-rot basidiomycetes and litter-decomposing fungi, tolerate this treatment and grow on creosote-treated wood. These fungi have also been shown to deplete and detoxify PAHs in contaminated soil [24–31].

The first studies on the potential of ligninolytic fungi for use in PAH biodegradation can be attributed to 1985, when Bumpus et al. [32] reported that the white-rot basidiomycete *Phanerochaete chrysosporium* partially degraded benzo[a]pyrene to carbon dioxide. Later, PAH degradation has been reported, among others, for the genera *Phanerochaete *[7, 13], *Trametes* [8], *Bjerkandera *[33], *Coriolus *[34], *Nematoloma* [18], *Irpex* [35], and *Pleurotus *[14–16, 21–23, 28, 30, 33, 36].

Different authors have associated the ability of these fungi to degrade PAHs with the extracellular ligninolytic system, which includes lignin peroxidase (LiP), Mn-peroxidase (MnP), versatile peroxidase (VP), and laccase (LAC) [8, 17, 37, 38]. Fungal laccases and peroxidases have been suggested to play a key role in lignin degradation and to enable their producers—the wood- and litter-decomposing fungi—to detoxify xenobiotic compounds by partial degradation or complete mineralization [35]. The catalytic action of these enzymes generates more polar and water-soluble metabolites, such as quinones, which are more susceptible to further degradation by indigenous bacteria present in soils and sediments [24]. Knowledge of all the metabolites formed from fungal metabolism is a key requirement to validate soil bioremediation. In soil, quinonic metabolites serve as substrates for microbial populations and are mineralized to carbon dioxide. They also may undergo polymerization and become part of the humus pool [27].

Several enzymatic mechanisms of PAH degradation by these fungi have been discussed: (a) LiP and MnP directly catalyze one-electron oxidation of PAHs with an ionization potential (IP) of 7.55 eV to produce PAH quinones [39–41], which can be further metabolized via ring fission [42]; (b) LAC catalyzes one-electron oxidation of PAHs, for example anthracene (ANT) and benzo[a]pyrene (B[a]P), whose efficiency is enhanced in the presence of mediators such as 1-hydroxybenzotriazole (HBT) or 2,2′-azinobis(3-ethylbenzthiazoline-6-sulfonic acid (ABTS) [43, 44]; (c) some PAH compounds containing up to six rings were shown to be degradable via MnP-dependent lipid peroxidation reactions both *in vitro* and *in vivo* [13, 25]; (d) intracellular cytochromeP-450 monooxygenase activity followed by epoxide hydrolase-catalyzed hydroxylation of 3-, 4-, and 5-ring PAHs are believed to initially metabolize PAH molecules, including phenanthrene (PHE), having an IP of 8.03 eV [14–16, 36, 45, 46].

In organisms that use the cytochrome P-450 system, the *trans*-dihydrodiol product cannot be used as an energy source, although further metabolism may occur. For example, B[a]P is oxidized by the cytochrome P-450 monooxygenase system; among other products, benzo[a]pyrene dihydrodiol epoxide is formed [46]. However, in white-rot fungi, such as *Phanerochaete chrysosporium* and *Trametes versicolor*, there occurs mineralization of PAHs, indicating the complete breakdown of PAHs [19]. At present time, substantial and conclusive evidence exists that ligninolytic enzymes are involved in PAH degradation by these fungi.

Two possible roles of ligninolytic system have been discussed up to now: (a) LiP, MnP, and LAC were found to have a pivotal role in the degradation of PAHs, catalyzing the first attack of molecule [10, 44]; (b) cytochrome P-450 monooxygenase could be responsible for this initial step [14–16, 46], and the ligninolytic mechanism was supposed to be involved in later steps of metabolism leading to CO_2_ evolution [16].

This paper summarizes the data available on PAH degradation by fungi belonging to different ecophysiological groups (white-rot and litter-decomposing fungi) under submerged cultivation and during mycoremediation of PAH-contaminated soils. The possible functions of ligninolytic enzymes of these fungi in PAH degradation are discussed.

## 2. PAH Degradation, the Key Products, and the Time Course of Ligninolytic Enzyme Production

### 2.1. Submerged Cultivation Conditions

Various publications have shown the ability of white-rot and litter-decomposing fungi to degrade different PAHs (Table 1), including phenanthrene (PHE) (by* T. versicolor, Kuehneromyces mutabilis *[9], *P. chrysosporium *[45], and *Pleurotus ostreatus* [14–16]), anthracene (ANT) (by *Bjerkandera* sp. BOS55 [47], *P. ostreatus *[14], *P. chrysosporium, T. versicolor, B. adusta *[12], and *I. lacteus *[48]), fluorene (FLU) (by *P. ostreatus *[14]), pyrene (PYR) (by *Bjerkandera* sp. BOS55 [47], *P. ostreatus *[14, 49], *Irpex lacteus *[48, 49], *T. versicolor* [49], and *P. chrysosporium* [49]), fluoranthene (FLA) (by *I. lacteus *[48], *T. versicolor, *and *Kuehneromyces mutabilis *[9]), chrysene (CHR) (by *P. ostreatus *[21]), benzo[a]pyrene (B[a]P) (by *Bjerkandera* sp. BOS55 [47], *P. chrysosporium, T. versicolor, *and *B. adusta *[12]), benz[a]anthracene (B[a]A) (by *I. lacteus *[50]), and dibenzothiophene (by *P. ostreatus *[14]).

In various studies, quinones have been identified as major products in the degradation of PAHs by fungi (Table 1) [12, 42, 51]. For example, PYR was metabolized by *P. ostreatus *predominantly to pyrene-4,5-dihydrodiol, ANT to antracene-1,2-dihydrodiol and 9,10-anthraquinone, FLU to 9-fluorenol and 9-fluorenone, and dibenzothiophene to corresponding sulfoxide and sulfone [14].

Many white-rot fungi can mineralize PAHs to CO_2_ as well. The PHE mineralization has been shown for *C. versicolor, I. lacteus *[52], *T. versicolor, *and *Kuehneromyces mutabilis *[9]. *C. versicolor *and* I. lacteus *also demonstrated high mineralization rates for 4-ring PYR [52]. The mineralization of PYR by *T. versicolor *and *Kuehneromyces mutabilis *was also found [9]. *P. ostreatus, *in addition to PYR [16, 49], mineralized B[a]P, ANT, and FLU but did not mineralize FLA [16].

#### 2.1.1. Phenanthrene

The most studied is the metabolism of PHE by *P. chrysosporium *[45, 53] and *P. ostreatus *[14–16]. In an early work Sutherland et al. [45] studied PHE degradation by *P. chrysosporium*; phenanthrene-*trans*-9,10-dihydrodiol, phenanthrene-*trans*-3,4-dihydrodiol, 9-phenanthrol, 3-phenanthrol, 4-phenanthrol, and the conjugate 9-phenanthryl-*D*-glucopyranoside were identified as metabolites. Since LiP was not detected in the culture medium, the authors suggested the involvement of monooxygenase and epoxide hydrolase activity in the initial oxidation and hydration of PHE by this fungus [45]. Dhawale et al. [53] showed that homokaryotic isolates of *P. chrysosporium* caused the disappearance of PHE when they were grown in low- as well as high-nitrogen media. Moreover, LiP and MnP activities were not detected in any of the cultures incubated in the presence of PHE. Additionally, mineralization of PHE was observed even under nonligninolytic conditions. The authors suggested that LiP and MnP are not essential for the degradation of PHE by *P. chrysosporium* [53]. 

Later, Song [52] found that the fungus *P. chrysosporium* oxidized PHE and phenanthrene-9,10-quinone at their C-9 and C-10 positions to give a ring-fission product, 2,2′-diphenic acid. 2,2′-diphenic acid formation from PHE was somewhat greater in low-nitrogen (ligninolytic) cultures than in high-nitrogen (nonligninolytic) cultures. The oxidation of phenanthrene-9,10-quinone to 2,2′-diphenic acid was unaffected by the level of nitrogen added, and it was significantly faster than the cleavage of PHE to 2,2′-diphenic acid. Phenanthrene-*trans*-9,10-dihydrodiol, previously shown to be the principal PHE metabolite in nonligninolytic *P. chrysosporium* cultures, was not formed in the ligninolytic cultures of this fungus. The authors suggested that PHE degradation by *P. chrysosporium* proceeds in the order PHE → phenanthrene-9,10-quinone → 2,2′-diphenic acid, involves both ligninolytic and nonligninolytic enzymes, and is not initiated by the classic microsomal cytochrome P-450. The extracellular LiP of *P. chrysosporium* was not able to oxidize PHE *in vitro,* and therefore, is also unlikely to catalyze the first step of PHE degradation *in vivo *[54].

Later, the important roles of both cytochrome P-450 and MnP in PHE metabolism by *P. chrysosporium *were found [55]. Ning et al. [55] showed that the microsomal P-450 degraded PHE with a NADPH-dependent activity. One of the major detectable metabolites of PHE in the ligninolytic cultures and microsomal fractions was identified as phenanthrene-*trans*-9,10-dihydrodiol. Piperonyl butoxide, a P-450 inhibitor that had no effect on MnP activity, significantly inhibited PHE degradation and *trans*-9,10-dihydrodiol formation in both intact cultures and microsomal fractions. Furthermore, PHE was also efficiently degraded by the extracellular fraction with high MnP activity. The authors suggested the involvement of both cytochrome P-450 and MnP in PHE metabolism by *P. chrysosporium *[55].

The other much studied white-rot fungus, *Pleurotus ostreatus*, when grown in basidiomycetes-rich medium, metabolized 94% of PHE added; 3% was mineralized to CO_2_. Approximately 52% of PHE was metabolized to *trans*-9,10-dihydroxy-9,10-dihydrophenanthrene (phenanthrene-*trans*-9,10-dihydrodiol), 2,2′-diphenic acid, and unidentified metabolites. ^18^O_2_-labeling experiments indicated that one atom of oxygen was incorporated into the phenanthrene-*trans*-9,10-dihydrodiol. Significantly less phenanthrene-*trans*-9,10-dihydrodiol was observed in incubations with cytochrome P-450 inhibitors. The experiments with cytochrome P-450 inhibitors and ^18^O_2_ labeling and the formation of phenanthrene-*trans*-9R,10R-dihydrodiol as the predominant metabolite suggested that *P. ostreatus* initially oxidizes PHE stereoselectively by a cytochrome P-450 monooxygenase and that this is followed by epoxide hydrolase-catalyzed hydration reactions [13].

Another white-rot fungus, *T. versicolor,* removed about 46% and 65% of PHE added in shaken and static cultures. PHE degradation was maximal at pH 6, and the optimal temperature was 30°C. Although the PHE removal percentage was highest (76.7%) at 10 mg/L of PHE, the transformation rate was maximal (0.82 mg/h) at 100 mg/L of PHE in the fungal culture [20]. *Agrocybe* sp. CU-43, a white-rot fungus isolated from Thailand, metabolized about 99% of 100 ppm PHE [57].

#### 2.1.2. Anthracene

The degradation of another 3-ring PAH, ANT, was found in most of the studied fungi. For example, *Agrocybe* sp. CU-43 removed about 92% of ANT [57]; *B. adusta, *38%; *P. ostreatus,* about 60% of these compounds [59]. All the tested white-rot fungi oxidized ANT to anthraquinone. The appearance of anthraquinone, coinciding with ANT degradation, is common to white-rot fungi as the first step [60]. Field et al. [12] concluded that anthraquinone behaves like a dead-end metabolite in certain white-rot fungi, including strains of the genera *Bjerkandera* and *Phanerochaete*. In liquid culture containing ANT, *I. lacteus *accumulated the degradation product anthraquinone [48]. However, four *Trametes* strains removed ANT without significant accumulation of the quinone; the ability of these fungi to metabolize anthraquinone was confirmed as well [12]. Anthraquinone did not accumulate in *P. ostreatus, Coriolopsis polyzona, *or *T. versicolor*, indicating that it was degraded further. *P. ostreatus *and *C. polyzona *failed to degrade anthraquinone in the absence of ANT [60]. Hammel et al. [42] showed also that the oxidation of ANT by *P. chrysosporium *to anthraquinone was rapid and that both compounds were significantly mineralized. Both compounds were cleaved by the fungus to give the same ring-fission metabolite, phthalic acid, and phthalate production from anthraquinone was shown to occur only under ligninolytic culture conditions. The results suggest that the major pathway for ANT degradation proceeds in the order ANT → 9,1-anthraquinone → phthalate → CO_2_ and that it is probably mediated by enzymes of ligninolytic metabolism [42].

#### 2.1.3. Fluorene

The degradation of FLU by *Agrocybe* sp. CU-43 [57], *B. adusta, *and *P. ostreatus* [59] was also found. Two of the metabolites from FLU degradation by *Agrocybe* sp. CU-43 were identified as 9-fluorenol and 9-fluorenone, the less toxic intermediates of FLU. However, 9-fluorenol is not an end product for the degradation [57].

#### 2.1.4. Pyrene

*I. lacteus *metabolized most of the added 4-ring PAH PYR; almost 50% of PYR was converted to polar metabolites and was recovered from an aqueous phase of the culture [52]. The accumulation of pyrene-4,5-dihydrodiol during PYR degradation in Kirk's medium by *P. ostreatus *D1 was found also [22]. However, in basidiomycetes-rich medium, the accumulation of pyrene-4,5-dihydrodiol by this fungus was not found, and PYR degradation was complete, with a PHE-derivative and phthalic acid being formed as intermediates. Phthalic acid, in turn, can be involved in basal metabolism [22].

#### 2.1.5. Chrysene

The same data were obtained for CHR bioconversion by the fungus *P. ostreatus* D1 [21]. In this case, the dependence of the completeness of CHR degradation on the cultivation conditions was found as well [21]. In Kirk's medium, accumulation of the quinone metabolite was found; in basidiomycetes-rich medium, no quinone was accumulated and CHR degradation was complete, with phthalic acid being formed as an intermediate [21].

#### 2.1.6. Benzo[a]Anthracene

Studies with B[a]A and its 7,12-dione indicated that only small amounts of quinone products were ever present in *Phanerochaete laevis* cultures and that quinone intermediates of PAH metabolism were degraded faster and more extensively by *P. laevis* than by *P. chrysosporium *[7]. *I. lacteus *incubated in a nutrient liquid medium degraded more than 70% of the initially applied B[a]A. At the first step of metabolization, B[a]A was transformed via a typical pathway of ligninolytic fungi to benz[a]anthracene-7,12-dione. The product was further transformed *via* at least two routes, one being similar to the ANT metabolic pathway of *I. lacteus. *Benz[a]anthracene-7,12-dione was degraded to 1,2-naphthalenedicarboxylic and phthalic acids, which was followed by production of 2-hydroxymethyl benzoic acid or monomethyl and dimethyl esters of phthalic acid. Another degradation product of benz[a]anthracene-7,12-dione was identified as 1-tetralone. Its transformation *via* 1,4-naphthalenedione, 1,4-naphthalene-diol and 1,2,3,4-tetrahydro-1-hydroxynaphthalene again resulted in phthalic acid. None of the intermediates were identified as dead-end metabolites [50].

#### 2.1.7. Benzo[a]Pyrene

First studies with B[a]P were conducted with *P. chrysosporium* and demonstrated B[a]P oxidation to quinones and partial mineralization of [^14^C]BaP. More recently, other fungi of these ecophysiological groups, such as *Bjerkandera* sp. BOS55 and *P. ostreatus*, were found to degrade B[a]P. The litter-decomposing basidiomycete *Stropharia coronilla*, which preferably colonizes grasslands, was found to be capable of metabolizing and mineralizing B[a]P in liquid culture [11]. In all cases, the degradation of B[a]P has been attributed to the activity of ligninolytic enzymes [11].

Finally, the nature of the transformation products formed during pollutant degradation differs among white-rot species (Table 1). This has been best demonstrated for ANT degradation. Significant accumulations of 9,10-anthraquinone were detected concomitant with depletion of ANT from liquid cultures of LAC-free species belonging to several genera (*Bjerkandera*, *Phanerochaete*, and *Ramaria*), with none or very little detected in LAC-producing species (*Trametes, Pleurotus,* and *Daedaleopsis*). LiP, MnP, VP, and LAC are all known to be produced by white-rot fungi, although the specific enzyme complements of different species are highly variable. Undoubtedly, all three levels of variability are consequences of both differences in the enzymology of the various white-rot species and differences in growth and enzyme production responses of various fungi to different culture media. Typical ligninolytic enzyme patterns of the white-rot fungi in liquid cultures are the following: *P. chrysosporium,* LiP and MnP; *P. ostreatus,* MnP, VP, and LAC; *T. versicolor,* LiP, MnP, and LAC. Each of these enzyme classes has been implicated in pollutant degradation by these fungi [7].

Summarizing the data presented above it should be noted that involvement of intracellular cytochrome P-450 in PAH degradation clearly showed only for PHE degradation [13, 45, 54, 55]. In many other cases, the degradation of PAHs has been attributed to the activity of ligninolytic enzymes [11, 21, 22, 42, 50]. The transport of PAHs inside fungal cell can be limited by solubility of these compounds. It is known that the solubility of PAHs in aqueous solutions is very low (0.003–1.3 mg/L) [4], and PHE is one from more soluble (1.3 mg/L). It can be suggested that the relatively well-soluble PHE can penetrate inside fungal cell where it is available to intracellular cytochrome P-450. However the less-soluble compounds cannot penetrate to fungal cell and should be metabolized firstly by extracellular enzymes.

#### 2.1.8. Ligninolytic Enzymes Production during PAH Degradation

Several studies have shown that diverse white-rot fungi are capable of PAH mineralization and that mineralization rates correlate with the production of ligninolytic enzymes [9, 12, 37]. It was found that PAH conversion was correlated with the production of MnP and LAC [63]. These enzymes have been repeatedly implicated in biodegradation of PAHs, including PHE [17, 25, 43, 44]. In *P*. *ostreatus *cultures, these enzymes were present at least during the first 3 weeks of the experiment, and thus their involvement in the removal of PAHs was possible, including the production of anthraquinone from ANT [43, 44]. In the case of *P. chrysosporium *and *C. polyzona, *ANT degradation to anthraquinone started earlier than the production of LiP but during the same time slot as the secretion of MnP [60].

Clear indications have been found that extracellular MnP is involved in the conversion process, since Mn^2+^ supplementation considerably stimulated both enzyme production and degradation of PAHs [10]. For example, Steffen et al. [10] showed that the litter-decomposing *Stropharia rugosoannulata* was the most efficient degrader, removing or transforming B[a]P almost completely, and about 95% of ANT and 85% of PYR, in cultures supplemented with 200 *μ*M Mn^2+^. In contrast, less than 40, 18, and 50% of B[a]P, ANT, and PYR, respectively, were degraded in the absence of supplemental Mn^2+^. In the case of *Stropharia coronilla*, the presence of Mn^2+^ led to a 20 fold increase in ANT conversion. The effect of manganese could be attributed to the stimulation of MnP. The Mn^2+^-supplemented cultures degraded about 6% of ^14^C-labeled B[a]P to ^14^CO_2_, whereas only 0.7% was mineralized in the absence of Mn^2+^ [10].

Mn^2+^ stimulated considerably both the conversion and the mineralization of B[a]P by the litter-decomposing basidiomycete *Stropharia coronilla*; the fungus metabolized and mineralized about four and twelve times, respectively, more of the B[a]P in the presence of supplemental Mn^2+^ than in the basal medium. This stimulating effect could be attributed to MnP, whose activity increased after the addition of Mn^2+^ [11].

Collins and Dobson [8] reported high MnP activities and substantial oxidation of PHE and FLU when liquid cultures of the white-rot fungus *T. versicolor *were supplemented with Mn^2+^. Despite the apparent reliance of the strain primarily on MnP, liquid cultures of *P. laevis* were capable of extensive transformation of ANT, PHE, B[a]A, and B[a]P. No LiP was found in the culture medium [7].

Two strains of *P. sordida *degraded a significantly greater amount of PHE and FLA than *P. chrysosporium. *The production of MnP, the only extracellular ligninolytic enzyme detected during the cultivation, was evaluated [56]. The fungus *I. lacteus *was shown to be an efficient degrader of PAHs possessing 3–6 aromatic rings. The strain produced mainly MnP in pollutant-free media. However, after contamination with PAHs (especially PYR), the values increased, and significant activity of Mn-independent peroxidase appeared in the complex medium [62].

LAC activity was also implicated in the degradation of PAHs by white-rot fungi [44]. A high and relatively stable activity of LAC was observed during degradation of ANT by *T. trogii *[61]. Hovewer, Han et al. [20] showed that LAC production by *T. versicolor* 951022 was not enhanced by addition of PHE. The addition of CuSO_4_, citric acid, gallic acid, tartaric acid, veratryl alcohol (VA), guaiacol, and ABTS enhanced the degradation of PHE and PYR and increased laccase activities in submerged culture of *Ganoderma lucidum* [58].

The effect of PAHs on the time course of LAC production by *P. ostreatus* D1 under conditions of submerged cultivation in Kirk's medium was also studied. It was shown that PHE, FLA, PYR, and CHR actively induced this enzyme, whereas FLU and ANT had a smaller effect [64].

Pozdnyakova et al. [23] showed that the activities of two ligninolytic enzymes, LAC and versatile peroxidase (VP), of *P. ostreatus* D1 were induced by the PAHs, their derivatives, and their degradation products under conditions of submerged cultivation in basidiomycetes rich medium. LAC was produced mostly in the first 7–10 days, whereas the production of VP began after 5–7 days of cultivation. The difference in the production time for these enzymes suggests that LAC can be involved in the first stages of PAH degradation and that VP can be necessary for oxidation of some degradation products. That was the first report on VP induction by PAHs and their derivatives [23].

Furthermore, LAC activity was revealed on the mycelial surface of *P. ostreatus* D1 cultivated in the presence of PYR and CHR [21, 22].

#### 2.1.9. Bioavailability of PAHs

As was mentioned above, the solubility of PAHs in aqueous solutions is very low. Bioavailability of PAHs may be the limiting factor for fungal and microbial attack [4]. In experimental conditions the addition of a detergent increases the solubility of PAHs and allows repeatable determination of the substrate and products. For increasing the substrate availability to ligninolytic enzymes and cells, nonionic surfactants such as Tween-20 and Tween-80 are usually used [47]. For example, various surfactants could increase the rate of ANT, PYR, and B[a]P oxidation by *Bjerkandera *sp. BOS55 by two-to-fivefold. The stimulating effect of surfactants was found to be solely due to the increased bioavailability of PAHs, indicating that the oxidation of PAHs by the extracellular ligninolytic enzymes is limited by low compound bioavailability [47].

At the present time, there are few reports on emulsifying compound production during PAH degradation by ligninolytic fungi. Song [49] showed that when *P. chrysosporium* was grown shaken, some foam could be seen, possibly because of production of a biosurfactant. The author suggested that this surfactant is responsible for the solubilization of pyrene in aqueous medium [49].

The production of an emulsifying agent during the degradation of phthalic, 2,2′-diphenic, and **α**-hydroxy-*β*-naphthoic acids, PHE, ANT, FLU, PYR, FLA, and CHR by the white-rot fungus *P. ostreatus* was found as well [65]. Emulsifying activity was inversely dependent on the water solubility of the compounds used. Maximal emulsifying activities were found in the presence of CHR (48.4%) and **α**-hydroxy-**β**-naphthoic acids (52.2%). The obtained data suggest that *P. ostreatus* D1 can produce some emulsifying agent as a response to the presence of PAHs and some products of their degradation and that the emulsifying agent can promote solubilization of these compounds [65]. Three different possible functions of the found emulsifying agent can be proposed: (a) this agent can be essential for increasing the solubility of hydrophobic compounds, (b) it could be involved in the oxidation of hydrophobic compounds catalyzed by ligninolytic enzymes [65], and (c) it can have a positive effect on the production of extracellular ligninolytic enzymes in agitated culture similar to that was described by Jager et al. [66].

The positive effect of an emulsifying agent on the production of extracellular ligninolytic enzymes in agitated culture was described by Jager et al. [66]. Those authors showed an increase in the production of LiP by *P. chrysosporium* in the presence of some detergents (Tween-80, Tween-20, and 3-[(3-colamidopropyl)dimethylammonio]1-propanesulfonate) [66]. The biosurfactant rhamnolipid increased the activities of LiP and MnP in *P. chrysosporium* and the activities of LiP and LAC in *Penicillium simplicissimum* [67].

The involvement of some surfactants in PAH oxidation by ligninolytic enzymes has been reported as well. For example, Böhmer et al. [68] showed PHE oxidation by the LAC/HBT pair in the presence of synthetic detergent (Tween-80) containing unsaturated lipids. They assumed that two coupling reactions take place: lipid peroxidation and PHE oxidation [68]. In line with the results of Böhmer et al., the data of Pozdnyakova et al. [69] showed that the *P. ostreatus* laccase/ABTS pair was able to oxidize PHE to a limited extent and that this reaction was greatly enhanced by Tween-80 [69]. Moen and Hammel [25] found a similar effect in a system containing *P. chrysosporium* MnP and Tween-80, and they showed coupling of the lipid peroxidation and PHE oxidation reactions [25]. 

Summarizing the data presented above it should be noted that the main studies of PAH degradation are carried out under different conditions (different medium and pH ligninolytic and nonligninolytic conditions) and with fungi, producing different sets of ligninolytic enzymes (e.g., *P. chrysosporium*: LiP and MnP, *T. versicolor*: LiP, MnP, LAC; and *P. ostreatus*: MnP, VP, LAC). It complicates the generalization and discussion of the data obtained by different authors. In my opinion, the same conditions of different fungi and correspondingly the different conditions of the same fungus can clarify the mechanisms of PAH degradation and the involvement of different group of enzymes. Now similar dependence of both bioconversion of PYR and CHR and ligninolytic enzyme production on the cultivation conditions was found for *P. ostreatus* D1 only [21, 22]. Under LAC production conditions, transformation of these PAHs occurred with accumulation of the quinone metabolites. Under both LAC and versatile peroxidase (VP) production conditions, CHR and PYR degradation occurred, with the stages leading to phthalic acid formation and its further utilization.

The second problem of PAH degradation which should be solved is bioavailability of these compounds. The study of natural emulsifying agent producing by ligninolytic fungi during xenobiotics degradation can promote the explanation of this problem.

Finally two groups of enzymes should be studied: cytochrome P-450 and cell-associated/intracellular forms of ligninolytic enzymes. The presence of many cytochrome P-450-related genes in white-rot fungi and some recent data on cytochrome P-450-catalyzed oxidation of PHE by *P. chrysosporium *[55] suggest the involvement of these enzymes in PAH degradation. However, our resent data [21, 22] suggest that the initial attack on the PAH molecule may be catalyzed by cell-associated enzymes (at least by LAC), because some time is required for the extracellular enzymes to appear in the culture medium at concentrations sufficient for substrate degradation.

### 2.2. Mycoremediation of PAH- and Oil-Contaminated Soils

Mycoremediation is a process by which fungi degrade or transform hazardous organic contaminants to less toxic compounds [70]. White-rot and litter-decomposing fungi are potential candidates for the treatment of contaminated soils because of their high capability to degrade a wide range of xenobiotics not only in liquid culture [10, 11, 71–76] but also in contaminated soil (Table 2) [72, 77–82]. Attempts have, therefore, been made to apply these fungi to the bioremediation of soils contaminated with compounds not sufficiently degradable by soil microorganisms [28].

For example, *L. lacteus* and *P. ostreatus *degraded three- and four-ring unsubstituted PAHs, including FLU, PHE, ANT, FLA, PYR, CHR, and B[a]A, in two contaminated industrial soils [80]. The biodegradation in soil by *P. chrysosporium *was approximately 80–85% of FLU, 52% of fluorenone, and 94% of 1,4-naphthoquinone [86]. It was found that the native soil microflora can be prompted into full mineralization of PAHs in some contaminated soils and that this mineralization can be enhanced when supplemented with the white-rot fungus *P. chrysosporium* [24]. The degradation of 4-ring PAHs including dibenzothiophene, fluoranthene, pyrene, and chrysene by *Bjerkandera *sp. BOS55 was shown too [82].

The degradation of PAHs in contaminated soil can also be attributed to the activity of ligninolytic enzymes. For example, periods of high *mnp* transcript levels and extractable MnP enzyme activity coincided with maximal rates of depletion of FLU and corresponding accumulation of 9-fluorenone, and CHR disappearance in soil cultures, supporting the hypothesis that PAHs are oxidized in soil via MnP-dependent mechanisms and that these reactions play a role in soil bioremediation by these fungi [26].

The removal efficiency of three-, four-, and five-ring PAHs in contaminated soil bioaugmented with *Anthracophyllum discolor* was investigated, and the production of lignin-degrading enzymes and PAH mineralization in the soil were also determined. A high removal capability for PHE (62%), ANT (73%), FLA (54%), PYR (60%), and B[a]P (75%) was observed in autoclaved soil inoculated with *A. discolor* in the absence of indigenous microorganisms, and it was associated with the production of MnP. The metabolites found in PAH degradation were anthraquinone, phthalic acid, 4-hydroxy-9-fluorenone, 9-fluorenone, and 4,5-dihydropyrene. *A. discolor* was able to mineralize 9% of PHE [84].

The effect of the indigenous soil microflora on growth, extracellular enzyme production, and PAH degradation efficiency in soil by the white-rot fungi *T. versicolor* and *I. lacteus* was investigated too. Both fungi were able to colonize soil. LAC was produced in *T. versicolor* cultures in the presence or absence of bacteria, but live bacteria decreased the LAC levels in soil by about 1/5. MnP was not detected in *T. versicolor *cultures. The amounts of MnP and LAC in *I. lacteus* cultures were not affected by the presence of bacteria. The rates of PAH removal by *T. versicolor* in sterile soil were 1.5, 5.8, and 1.8 fold for 2-3-ring, 4-ring, and 5-6-ring PAHs, compared to *I. lacteus*, respectively. *I. lacteus* showed a low efficiency of removal of PYR, B[a]A, and benzo[k]fluoranthene, compared to *T. versicolor*, whereas CHR and benzo[b]fluoranthene were degraded by neither fungus. Weak fungal/bacterial synergistic effects were observed in the case of removal of acenapthylene, B[a]P, dibenzo[a,h]anthracene and benzo[g,h,i]perylene by *I. lacteus* and acenaphthylene by *T. versicolor* [85].

Initially, the potential of *P. ostreatus *for mycoremediation of PAH-contaminated soil was evaluated in a model system. For example, the degradation of eight unlabeled highly condensed PAHs and the mineralization of three ^14^C-labeled PAHs by the white-rot fungus *Pleurotus* sp. Florida were investigated in sterile sea sand. PAH-loaded sea sand was mixed into a straw substrate and incubated. The disappearance of the unlabeled four-to-six-ring PAHs, including PYR, B[a]A, CHR, benzo[b]fluoranthene, benzo[k]fluoranthene, B[a]P, dibenzo[a,h]anthracene, and benzo[ghi]perylene, was determined. *Pleurotus* sp. Florida mineralized 53% of [^14^C]PYR, 25% of [^14^C]B[a]A, and 39% of [^14^C]B[a]P to ^14^CO_2_ in the presence of eight unlabeled PAHs (50 lg applied) within 15 weeks [87].

Later, Zebulun et al. [88] showed that after 90 days of incubation of *P. ostreatus *in soil, the level of contamination decreased, and fungal treatment affected the rate of degradation of all levels of ANT contamination. The inoculated soil showed more degradation of ANT (76–89%) than did the control soil. The release of ligninolytic peroxidases and LAC by *P. ostreatus* was associated with the observed ANT degradation [91]. 

The effect of salinity of the soil on PAH degradation and ligninolytic enzymes production was studied by Valentín et al. [81]. The minimal effect of salinity on ligninolytic activities of *I. lacteus *and *Lentinus tigrinus *was shown, while in *B. adusta, *activity was inhibited by salinity level of 32% [81].

A consortium of three basidiomycetes, including *T. versicolor*, *B. adusta*, and *B. fumosa*, grown on straw was found able to efficiently colonize soil and remove about 56 out of 100 mg/kg of soil dry weight of PYR in 28 days; in the mean time, the germination index increased, indicating a reduction in phytotoxicity. Enzymatic assays showed that LAC and manganese-independent peroxidase activity could have played a role in the degradation process [90].

The effects of concomitant pollutants, such as heavy metals, on PAH degradation in soil was evaluated by Baldrian et al. [30]. It was shown that *P. ostreatus* was able to degrade B[a]A, CHR, benzo[b]fluoranthene, benzo[k]fluoranthene, B[a]P, dibenzo[a,h]anthracene, and benzo[ghi]perylene in nonsterile soil both in the presence and in the absence of cadmium and mercury. During 15 weeks of incubation, recovery of individual compounds was 16 to 69% in soil without additional metals. While soil microflora contributed mostly to degradation of PYR (82%) and B[a]A (41%), the fungus enhanced the disappearance of less soluble PAHs containing five or six aromatic rings. Although the heavy metals in the soil affected the activity of ligninolytic enzymes produced by the fungus (LAC and MnP), no decrease in PAH degradation was found in soil containing Cd or Hg at 10 to 100 ppm. In the presence of cadmium at 500 ppm in soil, degradation of PAHs by the soil microflora was not affected, whereas the contribution of the fungus was negligible, probably owing to the absence of MnP activity. In the presence of Hg at 50 to 100 ppm or Cd at 100 to 500 ppm, the extent of soil colonization by the fungus was limited [30].

With the focus on alternative microbes for soil bioremediation, eight species of litter-decomposing basidiomycetous fungi were selected for a bioremediation experiment with an artificially contaminated soil. Up to 70%, 86%, and 84% of B[a]A, B[a]P, and dibenzo[a,h]anthracene, respectively, were removed in the presence of fungi, while the indigenous microorganisms converted merely up to 29%, 26%, and 43% of these compounds in 30 days. The low-molecular-mass PAHs studied were easily degraded by the soil microbes, and only ANT degradation was enhanced by the fungi as well. The agaric basidiomycetes *Stropharia rugosoannulata* and *Stropharia coronilla* were the most efficient PAH degraders among the litter-decomposing species used [83].

Similar to what is observed liquid medium, the mineralization of PAHs during mycoremediation of contaminated soil occurs also. For example, the mineralization of ^14^C-PYR in sterilized and nonsterile soil was investigated by using the wood-decaying fungi *Kuehneromyces mutabilis *and *Agrocybe aegerita. *In sterile soil, 5.1% and 1.5% of PYR was mineralized to ^14^CO_2_ by *K. mutabilis *and *A. aegerita*, respectively. During soil inoculation with the fungi, the mineralization was higher (47.7% for *K. mutabilis *and 38.5% for *A. aegerita*). In comparison with the indigenous microflora, *K. mutabilis *enhanced PYR elimination up to 42% [92].

Compared to wood, soil and litter are a more complex and heterogeneous environment, which may hamper the detection and estimation of enzyme activities. LAC activity reflects the course of degradation of organic substances, and thus it varies with time. Being the most abundant ligninolytic enzyme in soil, LAC also reflects the presence of fungal mycelia and participates in the transformation of lignin in forest litter. It is also generally presumed that LAC is able to react with soil humic substances that can be directly formed from lignin [89]. 

Novotný et al. [93] compared the abilities of *P. chrysosporium*, *P. ostreatus*, and *T. versicolor *to degrade PAHs and produce ligninolytic enzymes in soil. They found that colonization of sterilized soilby straw-grown inocula and degradation of ANT, PHE, and PYR were greatest with *P. ostreatus. *The production of MnP and LAC in soil was similar for *P. ostreatus *and *T. versicolor *but was extremely low for *P. chrysosporium. I. lacteus *efficiently colonized sterile and nonsterile soil by mycelium growing from a wheat straw inoculum. Good colonization of nonsterile gasworks soil contaminated with PAHs and heavy metals was also observed. *I. lacteus *efficiently removed three- and four-ring PAHs, including ANT, FLA, and PYR, from artificially spiked soil. LiP and LAC, but not MnP, were also detected when the fungus colonized the soil [48].

The relationship between ligninolytic activity and PAH degradation by *P. ostreatus *was demonstrated by Eggen [29]. In *P. ostreatus*, LAC and MnP were found, and their involvement in the removal of PAHs was possible, including the production of anthraquinone from ANT [93]. A similar correlation also was reported for the expression of MnP and the removal of FLU and CHR by soil cultures of *P. chrysosporium *[26].

The production of ligninolytic enzymes during mycoremediation of old oil-contaminated soil by 12 strains of white-rot fungi was studied by Pozdnyakova et al. [31]. LAC activity peaked during the first 2 weeks after sunflower-seed hulls colonized by the fungi had been added to the soil. Thereafter, LAC activity decreased and reached a minimal value, which was maintained in the course of the experiment. Enzyme activities in sterile and nonsterile soil were similar, but LAC activity tended to be higher in sterile soil. The fungi that were good colonizers of contaminated soil were good producers of LAC under the conditions used. The most active producers of LAC under these conditions were *Agaricus* sp. and *P. ostreatus *strains. Only *Pleurotus *and *Agaricus *strains produced peroxidase under these conditions. In either case, peroxidase activity was low and, as in the case of LAC activity, was similar in sterile and nonsterile soil. It should be noted that the peroxidase activities of the *P. ostreatus *strains detected in the presence of Mn^2+^ exceeded those without Mn^2+^ by about 20% only. The peroxidase activities of the *Agaricus *sp. strains could be detected only in the presence of Mn^2+^ [31]. The obtained results demonstrate different production of ligninolytic enzymes with respect to growth yields of various white-rot fungi growing in soil. The growth rates, the mycelium densities, the production of ligninolytic enzymes, and the degradation of old oil-contamination decreased in the order *Agaricus *sp. > *P. ostreatus *> *L. edodes *> *Coriolus *sp. The strains of the white-rot fungus *P. ostreatus *and the litter-decomposing fungus *Agaricus *sp. were the most active producers of ligninolytic enzymes and the most active degraders of old oil-contamination in soil [31].

In summary, it should be noted that white-rot and litter-decomposing fungi can actively degrade PAHs as in a model soil systems as in the conditions of the real soil (Table 2), for example, in soils with high salinity and in soils containing concomitant contamination such as heavy metals. The detection of the metabolites, which are similar to submerged cultures (e.g., anthraquinone, 9-fluorenone, 4,5-dihydropyrene, and other), and production of the same ligninolytic enzymes suggest the using the common mechanisms of PAH degradation. The decrease of the toxicity of the soil during mycoremediation makes this method perspective for use.

## 3. Enzymology of PAH Degradation

White-rot fungi produce four major groups of enzymes for the degradation of lignin: lignin peroxidase (known as ligninase in early publications; LiP; EC 1.11.1.14), manganese-dependent peroxidase (manganese peroxidase, MnP; EC 1.11.1.13), versatile peroxidase (VP; EC 1.11.1.16), and laccase (LAC; EC 1.10.3.2) [106].

It is generally known that ligninolytic enzymes, that is, LiP, MnP, and LAC, are also involved in the degradation of a wide range of organopollutants, including PAHs [33, 39]. For example, Vyas et al. [60] proposed that *in vitro *degradation of ANT by a crude enzyme preparation of white-rot fungi attests to the involvement of ligninolytic enzymes in the oxidation of ANT to anthraquinone. Sanglard et al. [107] showed that LiP is responsible for the initial steps in B[a]P metabolism by *P. chrysosporium. *Crude extracellular peroxidases from *P. laevis* transformed ANT, PHE, B[a]A, and B[a]P either in MnP-Mn^2+^ reactions or in MnP-based lipid peroxidation systems. No transformation of B[a]A or PHE was observed. In contrast, MnP-dependent lipid peroxidation reactions supported transformation of all four PAHs [7]. Experiments with purified cell-free enzyme extracts have confirmed the role of ligninolytic enzymes in the attack on PAHs. Extracellular preparations of LiP from *P. chrysosporium* were among the first to be shown as capable of PAH oxidation [39, 51].

The exact role of individual ligninolytic enzymes in degradation is still unclear. One possible way of elucidating the participation in degradation is by performing degradation with isolated enzymes together with identification of degradation products (Table 3) [35]. Since LiP, MnP, and LAC appear to be the predominant ligninolytic enzymes produced during PAH metabolism, most research has focused on these enzymes [33].

### 3.1. Lignin Peroxidase

Lignin peroxidases [LiP; EC 1.11.1.14, 1,2-bis(3,4-dimethoxyphenyl)propane-1,3-diol: hydrogen peroxide oxidoreductase] catalyze the H_2_O_2_-dependent oxidative depolymerization of lignin. The overall reaction is represented by 1,2-bis(3,4-dimethoxyphenyl)propane-1,3-diol + H_2_O_2_ *↔* 3,4-dimethoxybenzaldehyde+1-(3,4-dimethoxyphenyl)ethane-1,2-diol + H_2_O. LiP is relatively nonspecific to its substrates and has been known to oxidize phenolic aromatic substrates and a variety of nonphenolic lignin model compounds as well as a range of organic compounds. LiPs have the unique ability to catalyze oxidative cleavage of carbon-carbon bonds and ether bonds (C–O–C) in nonphenolic aromatic substrates of high redox potential.

The enzyme activity of LiP is conveniently measured by the oxidation of veratryl alcohol (VA), the favored LiP substrate, to veratraldehyde by the increase in absorbance at 310 nm [106].

As the first of these enzymes, purified LiP from *P. chrysosporium* was shown to attack B[a]P via one-electron abstractions, leading to unstable B[a]P radicals that undergo further spontaneous reactions to hydroxylated metabolites and several B[a]P quinones [51]. Three products of B[a]P oxidation by LiP of *P. chrysosporium*, namely, benzo[a]pyrene-1,6-, 3,6-, and 6,12-quinones, were found. Simultaneously with the appearance of oxidation products, LiP was inactivated. As all peroxidases, LiP is inactivated by the presence of hydrogen peroxide [108]; the enzyme could be stabilized by the addition of VA to the reaction mixture. Addition of VA to the reaction mixture increased the oxidation rate by about 15 times, and the enzyme retained most of its activity during the B[a]P oxidation [51].

The majority of studies of LiP-catalyzed oxidation of PAHs have been done with LiP from *P. chrysosporium *(Table 3). For example, the oxidation of another PAH, ANT, by LiP of *P. chrysosporium *was described [39, 40, 51]. Anthraquinone was identified as the main product of ANT oxidation by LiP from *P. chrysosporium *[40]. Hammel et al. [39] showed that LiP of *P. chrysosporium* catalyzes the oxidation of certain PAHs with ionization potentials (IP) of <7.55 eV. This result demonstrates that the H_2_O_2_-oxidized states of LiP are more oxidizing than the analogous states of classical peroxidases. Experiments with PYR as the substrate showed that pyrene-1,6-dione and pyrene-1,8-dione are the major oxidation products. Gas chromatography/mass spectrometry analysis of LiP-catalyzed PYR oxidation done in the presence of H_2_O_2_ showed that the quinone oxygens come from water. The resulting quinones were not substrates for LiP. The one-electron oxidative mechanism of LiP applies not only to lignin and lignin-related substructures but also to certain polycyclic aromatic and heteroaromatic pollutants. The oxidation of PYR by whole cultures of *P. chrysosporium* also resulted in these quinones. The authors suggested that LiP is thus likely to catalyze the first step in the mineralization of these compounds by whole cultures of *P. chrysosporium *[39].

Vazquez-Duhalt et al. [41] used LiP from *P. chrysosporium* to study the oxidation of ANT, 1-, 2-, and 9-methylanthracenes, acenaphthene, FLA, PYR, carbazole, and dibenzothiophene. Of the compounds studied, LiP was able to oxidize those with IP of <8 eV. The highest specific activity toward PAHs was found in systems with pHs between 3.5 and 4.0. The reaction products contain hydroxyl and keto groups. The product of ANT oxidation was thought to be 9,10-anthraquinone. The products of LiP oxidation of 1- and 2-methylanthracene were 1- and 2-methylanthraquinone, respectively.

Three products were detected by these authors [41] from the oxidation of 9-methylanthracene: 9,10-anthraquinone, 9-methyleneanthranone, and 9-methanol-9,10-dihydroanthracene. In this case, carbon-carbon bond cleavage, yielding anthraquinone from 9-methylanthracene, was detected. The mass spectra of the two products from acenaphthene matched those of 1-acenaphthenone and 1-acenaphthenol. The product of dibenzothiophene oxidation by LiP was its sulfoxide, as determined by comparing the GC-mass spectrometry analysis with a sample of authentic dibenzothiophene sulfoxide. The UV spectrum of the product of PYR oxidation most closely matched that of 1,8-pyrenedione. Although FLA and carbazole were oxidized, their products were not identified [41].

Torres et al. [1] tested LiP, cytochrome *c*, and hemoglobin for oxidation of PAHs in the presence of hydrogen peroxide and showed that LiP oxidized ANT, 2-methylanthracene, 9-hexylanthracene, PYR, acenaphthene, and B[a]P; the unreacted compounds included CHR, PHE, naphthalene, triphenylene, biphenyl, and dibenzofuran. The oxidation of the aromatic compounds by LiP correlated with their IPs; only those compounds that had IPs of <8 eV were transformed. It was found that the reaction products from all three hemoproteins (LiP, cytochrome *c*, and hemoglobin) were mainly quinones, suggesting the same oxidation mechanism for the three biocatalysts. The product from ANT was anthraquinone, and the product from 2-methylanthracene was 2-methylanthraquinone. The oxidation products for PYR and B[a]P were pyrenedione and benzo[a]pyrenedione, respectively. The mass spectra of the two products from acenaphthene oxidation with LiP matched 1-acenaphthenone and 1-acenaphthenol [1].

### 3.2. Mn-Peroxidase

Manganese peroxidase [MnP; EC 1.11.1.13] is a heme-containing glycoprotein that requires hydrogen peroxide (H_2_O_2_) as an oxidant. MnP oxidizes Mn^2+^ to Mn^3+^, which then oxidizes phenolic rings to phenoxy radicals, leading finally to the decomposition of compounds. Owing to its high reactivity, Mn^3+^ has to be stabilized via chelation by dicarboxylic acids, such as malonate or lactate. In addition to phenolic structures, the MnP system has been reported to catalyze cleavage of nonphenolic lignin model compounds [18, 106].

Because LiP is not produced by all white-rot fungi, more recent studies have focused on MnP, which is wide spread among the basidiomycetes and is found not only in wood-decay fungi but also in litter-decomposing fungi (Table 3). PAH degradation by MnP was first described in *P. chrysosporium *as a lipid peroxidation-dependent process [25]. Later, it was demonstrated that PAH degradation by some MnPs also occurs directly. PAH degradation experiments showed that MnP from *I. lacteus *was able to efficiently degrade three- and four-ring PAHs, including PHE, ANT, FLA, and PYR; PHE and FLA have IPs higher than 7.8 eV. The major degradation products of ANT were identified [35]. MnP produced by *Anthracophyllum discolor*, a Chilean white-rot fungus, degraded PYR (>86%), and ANT (>65%) alone or in mixture, and, to a lesser extent, it degraded FLA (<15.2%) and PHE (<8.6%) [94].

MnP-catalyzed oxidation of PAHs resulted in corresponding quinones. For example, MnP from *P. chrysosporium *oxidized ANT to anthraquinone [40]. During the degradation of ANT by MnP, the formation of anthrone was also detected, which was an expected intermediate, and it was followed by the appearance of 9,10-anthraquinone. Anthraquinone has earlier been described as the common oxidation product in *in vitro* reactions of peroxidases. Further oxidation resulted in the formation of phthalic acid, as it was observed in ligninolytic cultures of *P. chrysosporium* [42]. The characteristic ring-cleavage product 2-(2′-hydroxybenzoyl)-benzoic acid indicates that MnP is even able to cleave the aromatic ring of a PAH molecule [25]. I found only a single report suggesting that MnP does not oxidize ANT in the presence of Mn^2+^ [41].

The high hydrophobicity of PAHs greatly hampers their degradation in liquid media. The enzymatic action of MnP in media containing a miscible organic solvent, acetone (36% v/v), was evaluated as a feasible system for the degradation of three PAHs: ANT, dibenzothiophene, and PYR. The order of degradability, in terms of degradation rates, was as follows: ANT > dibenzothiophene > PYR. ANT was degraded to phthalic acid. A ring cleavage product of the oxidation of dibenzothiophene, 4-methoxybenzoic acid, was also detected [95].

The first description of direct enzymatic mineralization of PAHs by MnPs indicates their important role in the oxidation of PAHs by wood-decaying fungi was showed by Sack et al. [37]. Degradation of PAHs by crude MnP preparation of *Nematoloma frowardii *(later been shown to be a *Phlebia* sp. [109]) was demonstrated for a mixture of eight different compounds (PHE, ANT, PYR, FLA, CHR, B[a]A, B[a]P, and benzo[b]fluoranthene) and for five individual PAHs, including PHE, ANT, PYR, FLA, and B[a]A. The oxidation of PAHs was enhanced by the addition of glutathione, a mediator substance, which is able to form reactive thiyl radicals. Glutathione-mediated MnP was capable of mineralizing ^14^C-PYR (7.3%), ^14^C-ANT (4.7%), ^14^C-B[a]P (4.0%), ^14^C-B[a]A (2.9%), and ^14^C-PHE (2.5%) [37]. The mineralization of B[a]P and ANT by MnP from the litter-decomposing fungus *Stropharia coronilla *was also shown by Steffen et al. [11]. The oxidation of B[a]P leads to B[a]P-1,6-quinone as a temporary intermediate [11].

The stimulatory effect of glutathione was demonstrated by Günther et al. [96], who reported the degradation of 30% of ANT and 12% of PYR by MnP from *N. frowardii *(later *Phlebia* sp. [109]). The addition of mediating agents, such as reduced glutathione, increased the oxidative strength of MnP; as a result ANT was completely reduced and 60% of PYR was degraded after 24 h. Crude MnP from the agaric white-rot fungus *N. frowardii *(*Phlebia* sp.) oxidized *in vitro *several ^14^C-PAHs, including PYR and B[a]P, leading to the formation of significant amounts of ^14^CO_2_ (“enzymatic combustion”); mineralization increased 3- to 10-fold when reduced glutathione (GSH) was present in the reaction solution. The GSH effect was attributed to the transient formation of particularly reactive thiyl radicals. However, it is rather unlikely that fungi secrete “valuable” substances such as GSH under natural conditions into their microhabitat [96].

Therefore, alternative redox mediators, enhancing the oxidative strength of the MnP system, have been sought. Very promising compounds acting as such mediators were found among the unsaturated fatty acids (e.g., oleic and linoleic acids) and their derivatives (e.g., Tween-80). These substances have been shown to act similarly to GSH and were detected in liquid and solid fungal cultures. In the presence of Tween-80, MnP was able to oxidize FLU, a PAH that cannot be directly oxidized by chelated Mn^3+^because of its high IP (8.2 eV) as well as a complex PAH mixture (creosote). Tween-80 enabled *Stropharia coronilla* MnP to convert a large amount of B[a]P into polar fragments, and BaP-1,6-quinone was detected as a transient metabolite, which was further broken down to unknown products [11]. *Stropharia coronilla* MnP oxidized the individual PAHs in a mixture of 16 different compounds according to their IP and the presence of Tween-80. Only B[a]P and ANT were oxidized by the simple MnP system, but the initiation of lipid peroxidation *via* unsaturated fatty acid components of Tween-80 resulted in a substantial decrease in the content of all other PAHs. In addition, it was reported that poorly bioavailable PAHs, such as the six-ring compound benzo[g,h,i]perylene, are also subject to MnP attack. Steffen et al. proposed that MnP is the key enzyme in the degradation of B[a]P and other PAHs by litter-decomposing basidiomycetes [10, 11].

It has previously been mentioned that PAH degradation by MnP was first described in *P. chrysosporium*as a lipid peroxidation-dependent process [25]. Compounds with up to six rings are degraded *in vitro* during MnP-dependent lipid peroxidation reactions, and these same compounds are depleted from liquid cultures of *P. chrysosporium* [13].

The oxidation of FLU, a PAH that is not a substrate for fungal LiP, was studied *in vitro* with *P. chrysosporium* extracellular enzymes. Oxidation of FLU to 9-fluorenone was obtained in a system that contained Mn^2+^, an unsaturated fatty acid, and either crude *P. chrysosporium* peroxidases or purified recombinant MnP. The oxidation of FLU was inhibited by the free-radical scavenger butylated hydroxytoluene but not by the LiP inhibitor NaVO_3_. Mn^3+^-malonic acid complexes could not oxidize FLU. Maximal formation of 9-fluorenone in this system required an unsaturated fatty acid, Mn^2+^, and crude MnP. These results indicate that FLU oxidation *in vitro* was a consequence of lipid peroxidation mediated by *P. chrysosporium* MnP [17].

Crude and purified MnP from *Stropharia coronilla* oxidized B[a]P efficiently, a process that was enhanced by the surfactant Tween-80. A clear indication was found that benzo[a]pyrene-1,6-quinone was formed as a transient metabolite, which disappeared over the further course of the reaction. Treatment of a mixture of 16 PAHs with MnP resulted in concentration decreases of 10 to 100% for the individual compounds, and again, a stimulating effect of Tween-80 was observed. Probably owing to their lower IP, poorly bioavailable, high-molecular-mass PAHs such as B[a]P, benzo[g,h,i]perylene, and indeno[1,2,3-c,d]pyrene were converted to larger extents than low-molecular-mass ones (e.g., PHE and FLU) [11].

The MnP of *P. chrysosporium* supported Mn^2+^-dependent, H_2_O_2_-independent lipid peroxidation, as shown by two findings: (a) linolenic acid was peroxidized to give products that reacted with thiobarbituric acid and (b) linoleic acid was peroxidized to give hexanal. MnP also supported the slow oxidation of PHE to 2,2′-diphenic acid in a reaction that required Mn^2+^, oxygen, and unsaturated lipids. It was shown that fungal peroxidases are associated with the plasma membrane, where they might initiate the peroxidation of the membrane lipids. Extracellular membranes are frequently attached to the hyphae of white-rot fungi, and peroxidases are found on these structures. The chemical composition of these extracellular membranes indicated that they contain lipids. An analysis of the extractable lipids in *P. chrysosporium* mycelium showed that they contain unsaturated fatty acids. Lipid peroxidation is frequently initiated by transition metal ions that react with endogenous lipid hydroperoxides, either oxidizing them to peroxy radicals or reducing them to alkoxy radicals. In the case of *P. chrysosporium,* possible initiators include Mn^2+^, Mn^3+^, and the peroxidase heme. The oxygen-centered radicals produced during lipid peroxidation are known to trigger xenobiotic cooxidations. PHE is most likely to be oxidized by the latter route, which would yield phenanthrene-9,10-quinone as an intermediate, which subsequently undergoes facile 9,10-cleavage to give 2,2′-diphenic acid [25].

### 3.3. Versatile Peroxidase

It has been reported that some MnPs isolated from the fungi *B. adusta*, *Bjerkandera* sp. strain BOS55, *Bjerkandera* sp. (B33/3), *B. fumosa*, *P. eryngii*, *P. ostreatus, *and *P. pulmonarius* exhibit activities on aromatic substrates similar to that of LiP. This group of enzymes, known as versatile peroxidases (VPs), is not only specific for Mn^2+^, as is MnP, but also oxidizes phenolic and nonphenolic substrates that are typical for LiP, including veratryl alcohol, methoxybenzenes, and lignin model compounds, in the absence of manganese [106].

There have been only a few reports on VP production during PAH degradation by white-rot fungi [21, 22]. Now I find only single report (Table 3) on the oxidation of PAHs by VPs [97]. Wang et al. studied the PAH oxidation by a purified MnLiP hybrid isoenzyme (in fact it is VP) from *B. adusta *in the presence and absence of manganous ions. The substrates were PAHs with ionization potentials of 7.43 eV or lower, including ANT, its methyl derivatives, PYR and B[a]P. The PAH metabolites were identified as the corresponding quinones [97].

The oxidation of 3- and 4-rings PAHs by VPs from *B. fumosa* and *P. ostreatus *D1 to the corresponding quinones was found by Pozdnyakova et al. too (unpublished data).

### 3.4. Laccase

Laccase (LAC; EC 1.10.3.2, benzenediol: oxygen oxidoreductase) belongs to a group of polyphenol oxidases containing copper atoms in the catalytic center and usually called multicopper oxidases. LAC catalyzes the reduction of oxygen to water accompanied by the oxidation of a substrate, typically a *p*-dihydroxy phenol or another phenolic compound. LACs have overlapping substrate specificity, which can be extended to non-phenolic aromatic compounds with the use of redox mediators. In the presence of some synthetic and natural mediators, LAC can oxidize such compounds as veratryl and benzyl alcohols, nonphenolic groups of the lignin polymer, and lignin model substances [89].

There have been many reports on PAH oxidation by purified fungal laccases (Table 3). Most such studies have been made with LACs of *T. versicolor*, *C. hirsutus, P. ostreatus*, and *Coriolopsis gallica*. For example, *T. versicolor *LAC, in combination with HBT, was able to oxidize two PAHs, acenaphthene and acenaphthylene; ABTS did not significantly influence the oxidation rate. LAC alone oxidized about 35% of the acenaphthene and only 3% of acenaphthylene. The main products detected after incubation were 1,2-acenaphthenedione and 1,8-naphthalic acid anhydride [102]. The purified LAC of *T. versicolor* did not transform PHE. The addition of a redox mediator, ABTS or HBT, to the reaction mixture increased the oxidation of PHE by LAC about 40% and 30%, respectively [20]. The *in vitro *oxidation of ANT and B[a]P, which have IPs of ≤7.45 eV, is catalyzed by LAC from *T. versicolor.* Oxidation of ANT was enhanced in the presence of ABTS, whereas ABTS was essential for the oxidation of B[a]P. Anthraquinone was identified as the major end product of ANT oxidation [44].

The oxidation of five PAHs, including ANT, B[a]P, FLA, PHE, and PYR, was catalyzed by LAC from *C. hirsutus* in the presence of the redox mediators ABTS and HBT. In the ABTS-mediated system, B[*α*]P was the most rapidly oxidized substrate, with ANT being the most rapidly oxidized in the HBT-mediated system. There was no clear relationship between the IP and the oxidation of the substrates. The degree of oxidation, by LAC of *C. hirsutus*, for the PAHs tested ranged from 10.9 to 97.2%. FLA and PYR were readily oxidized by *C. hirsutus* LAC in the presence of all the redox mediators used, ranging from 37.9 to 92.7%. PYR, one of the least oxidizable PAHs, was still oxidized by about 40% in the presence of all the mediators. From this, it was concluded that the PAH-oxidizing abilities of LAC are different, depending on the fungal species from which it was obtained [34].

The biotransformation of B[a]P by purified LAC of *Pycnoporus cinnabarinus *was shown, with the reaction requiring the presence of the exogenous mediator ABTS. Most of the substrate (95%) was converted within 24 hours. The enzyme oxidized the substrate mainly to benzo[a]pyrene-1,6-, 3,6- and 6,12-quinones at a 2/1/1 ratio [101].

LAC of the white-rot fungus *Ganoderma lucidum* degraded ANT completely without a redox mediator and also degraded B[a]P, FLU, acenapthene, acenaphthylene, and B[a]A up to 100.0, 98.6, 95.4, 90.1, and 85.3%, respectively, when the mediator was present [99].

Majcherczyk et al. [103] demonstrated that LAC of *T. versicolor* was able to oxidize *in vitro* most of the 14 PAHs tested. Acenaphthylene was removed by 37%, followed by ANT and B[a]P, which were oxidized by 18 and 19%, respectively. Lower but significant oxidation of about 10% was found for eight additional PAHs: acenaphthene, FLA, PYR, B[a]A, CHR, benzo[b]fluoranthene, benzo[k]fluoranthene, and perylene. Naphthalene, FLU, and PHE were recovered unchanged after incubation for 72 h with laccase. Addition of HBT to the reaction mixture increased the oxidation of PAHs: acenaphthylene, acenaphthene, FLU, ANT, B[a]P, and perylene were almost completely removed from the reaction mixture. Oxidation of PYR and B[a]A increased from 8 and 6% without a mediator to 48 and 53% in the presence of HBT. PAH-quinones as oxidation products were formed from all PAHs to different extents. Some of the PAHs were polymerized in the LAC/mediator system to products of average molecular weight (MW) of approximately 1,500 Da [103].

The effect of different mediators on LAC oxidation was studied by Pickard et al. [19]. The following seven PAHs were oxidized by LAC of *Coriolopsis gallica *UAMH 8260: B[a]P, 9-methylanthracene, 2-methylanthracene, ANT, biphenylene, acenaphthene, and PHE. 9-Methylanthracene was the substrate that was the most rapidly oxidized. There was no clear relationship between the ionization potential of the substrate and the first-order rate constant for substrate loss *in vitro* in the presence of ABTS. The effects of mediating substrates were examined further by using ANT as a substrate. HBT supported approximately one-half ANT oxidation rate that ABTS supported, but HBT plus ABTS increased the oxidation rate nine-fold, compared with the oxidation rate in the presence of ABTS. A synergistic effect of the two mediators was found [19].

LAC of the white-rot fungus *P. ostreatus* D1 was able to degrade ANT (91%), PHE (72%), FLU (53.5%), PYR (65.5%), FLA (69.7%), and perylene (73%) only in the presence of a synthetic mediator. The degradation of PHE in the presence of detergents varied from 49 to 72%, whereas in the absence of any detergent, it reached 10%. Investigating the effect of various mediators on PAH degradation showed that ABTS was a better mediator of ANT oxidation and that HBT was a better mediator of FLU oxidation. PYR and ANT were degraded more rapidly in a mixture than separately. The degradation yield depends on the structure of the PAH molecule, type of the organic solvent, the presence and type of a detergent, enzyme concentration, and duration of the reaction. It does not correlate with the IP values, solubility, and recalcitrance of the studied PAHs. Apparently, it is necessary to take into account all these factors for studies of the catalytic mechanism responsible for LAC-catalyzed oxidation of PAHs [69].

LAC from *Coriolopsis gallica *oxidized not only FLU (75%) but also its polycyclic heterocyclic analogs such as carbazole (100% loss), *N*-ethylcarbazole (100%), and dibenzothiophene (60%) in the presence of HBT and ABTS as free radical mediators. Susceptibility to LAC oxidation appears related to the ionization potential of the substrate: the compounds with an IP of above 8.52, namely, dibenzofuran (IP = 8.77) and benzothiophene (IP = 8.73) were not attacked. Carbazole (IP = 7.68) was the most sensitive to oxidation, with >99% being transformed after 1 h. 9-fluorenone was identified as the product of FLU (IP = 8.52) oxidation, and dibenzothiophene sulfone as product of dibenzothiophene (IP = 8.44) was found [98].

PHE was efficiently oxidized by LAC in the presence of both HBT and unsaturated lipids, with 73% of the initially added PHE being degraded. The system was also able to peroxidize linoleic acid to its corresponding hydroperoxides, suggesting the involvement of lipid peroxidation in LAC-catalyzed PHE oxidation. Lipid peroxidation by LAC required HBT and did not depend on Mn^2+^ or H_2_O_2_, suggesting that the chemical reactions involved differ from those previously reported for MnP. LAC efficiently oxidized PHE in the presence of HBT and Tween-80. Two major products were formed 2,2′-diphenic acid and phenanthrene-9,10-quinone. In contrast to HBT, neither ABTS nor 3-hydroxyanthranilic acid stimulated PHE oxidation under the experimental conditions used. This finding was not further investigated, but it seems likely that HBT forms more reactive intermediates than ABTS and 3-hydroxyanthranilic acid do when oxidized by LAC. PHE was poorly degraded when Tween-80 was replaced by Tween-20. Tween-80 contains unsaturated fatty acid esters, whereas Tween-20 does not. The results show that the LAC/HBT couple was able to oxidize PHE to a limited extent, and that this reaction was greatly enhanced by unsaturated lipids [68].

The white-rot fungi secrete a large number of low-molecular-weight aromatic compounds, some of which are phenol derivatives and potential LAC substrates. The oxidation of PAHs was studied by Cañas et al. [104] in systems consisting of LAC from *T. versicolor* and compounds known as mediator compounds. The enzymatic oxidation of acenaphthene, acenaphthylene, ANT, and FLU was mediated by various LAC substrates (phenols and aromatic amines) or compounds produced and secreted by white-rot fungi. The best natural mediators, such as phenol, aniline, 4-hydroxybenzoic acid, and 4-hydroxybenzyl alcohol were as efficient as the previously described synthetic compounds ABTS and HBT. The oxidation efficiency increased proportionally with the redox potentials of the phenolic mediators up to a maximum value of 0.9 V and decreased thereafter with redox potentials exceeding this value.

Natural compounds such as methionine, cysteine, and reduced glutathione, containing sulfhydryl groups, were also active as mediator compounds [105]. Efficient transformation of several PAHs was obtained by using a fungal LAC in the presence of phenolic compounds related to those formed in nature during the turnover of lignin and humus. The effect of these natural mediators, namely, vanillin, acetovanillone, acetosyringone, syringaldehyde, 2,4,6-trimethylphenol, *p*-coumaric acid, ferulic acid, and sinapic acid, was compared with that of synthetic mediators such as ABTS and HBT. ANT was significantly degraded by LAC in the absence of mediators, whereas B[a]P and PYR were weakly transformed (less than 15% after 24 h). Vanillin, acetovanillone, 2,4,6-trimethylphenol, and, above all, *p*-coumaric acid strongly promoted the removal of PAHs by LAC. 9,10-Anthraquinone was the main product detected from ANT oxidation by all the LAC mediator systems. The yield of anthraquinone formed was directly correlated with the amount of *p*-coumaric acid used. This compound resulted in a better LAC mediator than ABTS and close similarity to HBT, attaining 95% removal of ANT and B[a]P and about 50% of PYR within 24 h. Benzo[a]pyrene-1,6-, 3,6-, and 6,12-quinones were produced during B[a]P oxidation with LAC and *p*-coumaric acid, HBT, or ABTS as mediators, although use of the latter mediator gave further oxidation products that were not produced by the two other systems [105].

During solid-state fermentation of a natural lignin-containing substrate, the white-rot fungi produce what is known as a yellow form of LAC. The active center of this enzyme is modified by lignin-degradation products. As a result of this modification, LAC gains the ability to catalyze the oxidation of nonphenolic compounds without addition of mediators [100]. The catalytic activity of the yellow LAC from *P. ostreatus* D1 toward PAHs, their derivatives, and anthracene-like synthetic dyes, was investigated. Yellow LAC did not catalyze the oxidation of the two-ring PAH naphthalene, but the naphthalene derivatives **α**- and **β**-naphthols, **α**-nitroso-**β**-naphthol, **α**-hydroxy-**β**-naphthoic acid, and **α**-naphthylamine were all good LAC substrates. Yellow LAC degraded all the PAHs containing from three to five rings, with the following efficiencies: 91% for ANT, 40% for PYR, 95% for FLU, 47% for FLA, 82% for PHE, and 100% for perylene. These efficiencies were higher than that observed for a blue LAC from the same fungus in both absence and presence of the typical synthetic mediators ABTS and HBT under the same experimental conditions. Yellow LAC oxidized a model mixture of PAHs and all the synthetic dyes. The same product of ANT oxidation and various unidentified products of FLU oxidation were observed in solutions of various solvents [100].

Comparison of the catalytic properties of ligninolytic enzymes suggests that the role of LiP in PAH degradation limited by narrow range of compounds according to their IP values. In my opinion, MnP (perhaps VP too) and LAC are the most important enzymes in PAH oxidation. Their catalytic possibilities are extended the following factors (a) the presence of some natural and synthetic compounds (e.g., gluthatione for MnP and LAC; ABTS for LAC); (b) the modification of the active centre of LAC during cultivation of a fungi on lignin-containing natural substrates as it occurs in case of yellow LACs; (c) the coupling of PAH oxidation and lipid peroxidation (MnP and LAC). Thus, MnP and LAC probably plays a more important role than simply that of the initial oxidation and production of quinones.

## 4. Conclusions

In summary, according to the presented data the following conclusions can be made.

All the studied white-rot and litter-decomposing fungi can metabolize and mineralize PAHs both in liquid medium and in soil. The initial products of degradation in the case of all the studied PAHs by all the studied fungi are the corresponding quinones, regardless of the cultivation conditions.

The accumulation or the subsequent metabolization of the quinonic products can be determined by (a) the species of the white-rot or litter-decomposing fungus and the composition of their ligninolytic enzyme system; (b) the cultivation conditions and the produced enzyme complex under these conditions.

Large size of molecule and the poor solubility can determine the nature of enzyme system involved in their first attack. This system should be extracellular and nonspecific. The ligninolytic enzyme system of white-rot and litter-decomposing fungi is sufficient for such requirements. All the studied ligninolytic enzymes, LiP, MnP, VP, and LAC, oxidized PAHs to the corresponding quinones. MnPs not only oxidize PAHs but can cleave the aromatic ring and mineralize the resulting products to CO_2_. The efficiency of PAH oxidation by ligninolytic enzymes can be improved by different natural factors, such as natural and synthetic mediators, and by coupling PAH oxidation and lipid peroxidation.

In addition, the poor solubility of these compounds can be surmount by emulsifying compounds production during PAH degradation. The production of an emulsifying agent suggests three possibilities: (a) this agent can be essential for increasing the solubility of hydrophobic compounds, (b) it can have a positive effect on the production of extracellular ligninolytic enzymes in agitated culture, and (c) it could be involved in the oxidation of hydrophobic compounds catalyzed by ligninolytic enzymes.

Summarizing the data of different authors, I propose that the ligninolytic enzyme system plays a key role in the initial step of PAH degradation by white-rot and litter-decomposing fungi. The resulting metabolites are more soluble and can be taken inside the cell, where different intracellular enzymes (e.g., cytochrome P-450) can act. The main contradiction is that PAH degradation occurs before extracellular enzyme production. This contradiction can be solved if one takes into account the presence of a mycelia surface-bound LAC pool, which may be involved in the initial stages of PAH degradation.

In my opinion, more attention should be given to studies on the fate of PAH metabolites outside and inside of fungal cell. The intracellular enzymes probably involved in the utilization of these metabolites should be studied carefully. In this connection, the study of the intracellular and/or cell surface-bound pool of ligninolytic enzymes seems very important.

## Figures and Tables

**Table 1:** PAH degradation under submerged cultivation conditions.

PAH	Fungus	Metabolites	Enzymes	References
PHE	*P. chrysosporium *(WRF)	PHE-*trans-*9,10-dihydrodiol; PHE-*trans-*3,4-dihydrodiol; 9-phenantrol, 3-phenanthrol; 4-phenanthrol; 9-phenanthryl-*D-*glucopyranoside	Monooxygenase; epoxide hydrolase	[45]
		CO_2_	ND	[53]
		PHE-9,10-quinone; 2,2′-diphenic acid	ND	[52]
		PHE-*trans*-9,10-dihydrodiol	Cytochrome P-450; MnP	[55]
	*P. sordida *(WRF)	ND	MnP	[56]
	*P. ostreatus *(WRF)	PHE-*trans*-9,10-dihydrodiol	Cytochrome P-450; epoxide hydrolase	[13]
	*T. versicolor *(WRF)	ND	ND	[20]
	*Agrocybe *sp. (WRF)	ND	ND	[57]
	*Ganoderma lucidum *(WRF)	ND	LAC	[58]

ANT	*Agrocybe *sp. (WRF)	9,10-anthraquinone	ND	[57]
	*B. adusta *(WRF)	9,10-anthraquinone	ND	[59]
	*P. ostreatus *(WRF)	9,10-anthraquinone	MnP; LAC	[43, 44, 59, 60]
	*I. lacteus* (WRF)	9,10-anthraquinone	ND	[48]
	*Trametes versicolor*	9,10-anthraquinone	ND	[12, 60]
	*Coriolopsis polyzona *(WRF)	9,10-anthraquinone	ND	[60]
	*P. chrysosporium *(WRF)	9,10-anthraquinone; phthalic acid; CO_2_	LiP; MnP	[42, 60]
	*Stropharia coronilla *(LDF)	ND	MnP	[10]
	*T. trogii *(WRF)	9,10-anthraquinone	LAC	[61]

FLU	*Agrocybe *sp. (WRF)	9-fluorenol; 9-fluorenone	ND	[57]
	*B. adusta *(WRF)	ND	ND	[59]
	*P. ostreatus *(WRF)	ND	ND	[59]

PYR	*I. lacteus *(WRF)	Quinonic metabolites	MnP; Mn-inhibited peroxidase	[52, 62]
	*P. ostreatus *(WRF)	PYR-4,5-dihydrodiol	LAC	[22]
		PYR-4,5-dihydrodiol; phthalic acid	LAC, VP	[22]
	*Ganoderma lucidum *(WRF)	ND	LAC	[58]

FLA	*P. sordida* (WRF)	ND	MnP	[56]

B[a]A	*P. laevis *(WRF)	Quinone metabolite	ND	[7]
	*P. chrysosporium *(WRF)	Quinone metabolite	ND	[7]
	*I. lacteus *(WRF)	B[a]A-7,12-dione; phthalic acid, 1,2-naphthalenedicarboxylic acid; 2-hydroxymethyl benzoic acid; mono- and di-methyl esters of phthalic acid; 1-tetralone; 1,4-naphthalenedione; 1,4-naphthalenediol; 1,2,3,4-tetrahydro-1-hydroxynaphthalene	ND	[50]

B[a]P	*P. chrysosporium *	Quinone metabolite; CO_2_	LiP; MnP	[11]
	*Bjerkandera *sp. (WRF)	Quinone metabolite	ND	[11]
	*P. ostreatus *(WRF)	Quinone metabolite	ND	[11]
	*Stropharia coronilla *(LDF)	Quinone metabolite; CO_2_	LiP; MnP	[11]
	*Stropharia rugosoannulata *(LDF)	CO_2_	MnP	[10]

**Table 2:** PAH degradation under mycoremediation.

Fungus	PAH	Metabolites	Enzymes	References
*Agrocybe aegerita*	PYR	CO_2_	ND	[83]

*Anthracophyllum discolor*	PHE; ANT; FLA; PYR; B[a]P	9,10-anthraquinone; phthalic acid; 4-hydroxy-9-fluorenone; 9-fluorenone; 4,5-dihydropyrene; CO_2_ from PHE	MnP	[84]

*Bjerkandera *sp.	FLA; PYR; CHR	ND	ND	[82]

*I. lacteus*	FLU; PHE; ANT; FLA; PYR; CHR; B[a]A; B[a]P; benzo[k]fluoranthene	ND	LiP; MnP; LAC	[48, 80, 85]

*Kuehneromyces mutabilis*	PYR	CO_2_	ND	[83]

*P. chrysosporium*	FLU; CHR; ANT; PHE; PYR	9-fluorenone	MnP	[26, 86]

*Pleurotus* sp. Florida	PYR; CHR; B[a]A; B[a]P; benzo[b]fluoranthene; benzo[k]fluoranthene; dibenzo[a,h]anthracene; benzo[ghi]perylene	CO_2_ from PYR; CHR; B[a]A; B[a]P	ND	[87]

*P. ostreatus*	FLU; PHE; ANT; FLA; PYR; CHR; B[a]A; B[a]P; benzo[b]fluoranthene; benzo[k]fluoranthene; dibenzo[a,h]anthracene; benzo[ghi]perylene	9,10-anthraquinone	MnP; LAC	[30, 80, 88, 89]

* Stropharia rugosoannulata*	B[a]A; B[a]P; dibenzo[a,h]anthracene	ND	ND	[90]

*Stropharia coronilla*	B[a]A; B[a]P; dibenzo [a,h]anthracene	ND	ND	[90]

*T. versicolor*	ANT; PHE; PYR	ND	MnP; LAC	[89]

Consortium: * T. versicolor * * B. adusta * * B. fumosa *	PYR	ND	LAC; Mn-independent peroxidase	[91]

**Table 3:** PAH oxidation by pure enzymes.

Enzyme	Fungus	PAH	Products	References
LiP	*P. chrysosporium*	B[a]P	B[a]P-1,6-quinone	[1, 51]
			B[a]P-3,6-quinone	
			B[a]P-6,12-quinone	
		ANT	9,10-anthraquinone	[1, 40, 41]
		PYR	PYR-1,6-dione; PYR-1,8-dione	[1, 39, 41]
		FLA	ND	[41]
		1-methylanthracene	1-methylanthraquinone	[41]
		2-methylanthracene	2-methylanthraquinone	[1, 41]
		9-methylanthracene	9-anthraquinone; 9-methyleneanthranone; 9-methanol-9,10-dihydroanthracene	[41]
		Acenaphthene	1-acenaphthenone; 1-acenaphthenol	[1, 41]
		Dibenzothiophene	dibenzothiophene sulfoxide	[41]

MnP	*Anthracophyllum discolor*	PYR; ANT; FLA; PHE	ND	[94]
	*I. lacteus*	PHE; ANT; FLA; PYR	9,10-anthraquinone	[35]
	*P. chrysosporium*	ANT	anthrone; 9,10-anthraquinone; 2-(2′-hydroxybenzoyl)-benzoic acid; phthalic acid	[25, 40, 42, 95]
		FLU	9-fluorenone	[17]
		PHE	PHE-9,10-quinone; 2,2′-diphenic acid	[25]
		dibenzothiophene	4-methoxybenzoic acid	[95]
	*Nematoloma frowardii *(*Phlebia *sp.)	PHE; ANT; PYR; FLA; CHR; B[a]A; B[a]P; benzo[b]fluoranthene	CO_2_ from PHE; ANT; PYR; B[a]A; B[a]P	[37, 96]
	*Stropharia coronilla*	ANT; B[a]P	9,10-anthraquinone; CO_2_; B[a]P-1,6-quinone	[10, 11]

	*B. adusta*	ANT; PYP; B[a]P	9,10-anthraquinone	[97]
VP	*B. fumosa*	ANT; PHE; FLU; PYR; CHR; FLA	9,10-anthraquinone; 9-fluorenone	Pozdnyakova et al., unpublished data
	*P. ostreatus *D1	ANT; PHE; FLU; PYR; CHR; FLA	9,10-anthraquinone; 9-fluorenone	

LAC	*C. hirsutus*	ANT; PHE; PYR; FLA; B[a]P	ND	[34]
	*Coriolopsis gallica*	B[a]P; ANT; PHE; FLU; 9-methylanthracene; 2-methylanthracene; Acenaphthene; carbazole; N-ethylcarbazole; Dibenzothiophene	9-fluorenone; dibenzothiophene sulfone	[19, 98]
	*Ganoderma lucidum*	ANT; FLU; B[a]A; B[a]P; Acenaphthene; Acenaphthylene	ND	[99]
	*P. ostreatus *	ANT; PHE; FLU; PYR; FLA; perylene	9,10-anthraquinone; 9-fluorenone	[69, 100]
	*Pycnoporus cinnabarinus*	B[a]P	B[a]P-1,6-quinone; B[a]P-3,6-quinone; B[a]P-6,12-quinone	[101]
	*T. versicolor*	Acenaphthene; PHE; ANT; Acenaphthylene, B[a]P; ANT; FLA; PYR; B[a]A; CHR; perylene; benzo[b]fluoranthene; benzo[k]fluoranthene; FLU	1,2-acenaphthenedione 1,8-naphthalic acid anhydride; 9,10-anthraquinone; PHE-9,10-quinone, 2,2′-diphenic acid; B[a]P-1,6-quinone; B[a]P-3,6-quinone; B[a]P-6,12-quinone	[20, 44, 68, 102–105]

## Abbreviations

*P*: *Phanerochaete*
*C*: *Coriolus*
*T*: *Trametes*
*P*: *Pleurotus*
*I*: *Irpex*
LiP: Lignin peroxidase
MnP: Mn-peroxidase
VP: Versatile peroxidase
LAC: Laccase
HBT: 1-hydroxybenzotriazole
ABTS: 2,2′-azinobis(3-ethylbenzthiazoline-6-sulfonic acid)
VA: 3,4-dimethoxybenzyl alcohol, veratryl alcohol
PAHs: Polycyclic aromatic hydrocarbons
ANT: Anthracene
PHE: Phenanthrene
FLU: Fluorene
PYR: Pyrene
FLA: Fluoranthene
CHR: Chrysene
B[a]P: Benzo[a]pyrene
B[a]A: Benzo[a]anthracene
IP: Ionization potential
WRF: White-rot fungi
LDF: Litter-decomposing fungi.
